# Rapid Quantitative Point-Of-Care Diagnostic Test for Post COVID-19 Vaccination Antibody Monitoring

**DOI:** 10.1128/spectrum.00396-22

**Published:** 2022-03-31

**Authors:** Maria E. Moeller, Frederik N. Engsig, Melanie Bade, Jeppe Fock, Pearlyn Pah, Anna Louise Soerensen, Didi Bang, Marco Donolato, Thomas Benfield

**Affiliations:** a Department of Infectious Diseases, Copenhagen University Hospital - Amager and Hvidovre, Hvidovre, Denmark; b BluSense Diagnostics ApS, Copenhagen, Denmark; c Department of Clinical Microbiology, Copenhagen University Hospital - Amager and Hvidovre, Hvidovre, Denmark; d Institute of Clinical Medicine, Faculty of Health and Human Sciences, University of Copenhagen, Copenhagen, Denmark; University of Mississippi Medical Center

**Keywords:** S protein trimer, SARS-CoV-2, immunomagnetic agglutination assay, point-of-care, rapid IgG-IgM-IgA combined test, vaccination

## Abstract

Point-of-care (POC) quantification of antibody responses against SARS-CoV-2 spike protein can enable decentralized monitoring of immune responses after infection or vaccination. We evaluated a novel POC microfluidic cartridge-based device (ViroTrack Sero COVID-19 Total Ab) for quantitative detection of total antibodies against SARS-CoV-2 spike trimeric spike protein compared to standard laboratory chemiluminescence (CLIA)-based tests. Antibody responses of 101 individuals were measured on capillary blood, venous whole blood, plasma, and diluted plasma samples directly on the POC. Results were available within 7 min. As the reference, plasma samples were analyzed on DiaSorin LIAISON XL CLIA analyzer using LIAISON SARS-CoV-2 IgM, LIAISON SARS-CoV-2 S1/S2 IgG, and LIAISON SARS-CoV-2 TrimericS IgG assays. The Spearman rank’s correlation coefficient between ViroTrack Sero COVID-19 Total Ab and LIAISON SARS-CoV-2 S1/S2 IgG and LIAISON SARS-CoV-2 TrimericS IgG assays was found to be 0.83 and 0.89, respectively. ViroTrack Sero COVID-19 Total Ab showed high correlation between the different matrixes. Agreement for determination of samples of >230 binding antibody units (BAU)/mL on POC and CLIA methods is estimated to be around 90%. ViroTrack Sero Covid Total Ab is a rapid and simple-to-use POC test with high sensitivity and correlation of numerical results expressed in BAU/mL compared to those of a commercial CLIA assay.

**IMPORTANCE** Serological testing is an important diagnostic support tool in the fight against COVID-19. So far, serological testing has been performed on either lateral flow assays, which perform only qualitatively and can be difficult for the individual to read, or standard laboratory assays, which are time- and resource-consuming. The purpose of the study was to evaluate the performance of a new POC microfluidic cartridge-based device based on immunomagnetic agglutination assay that can provide an accurate numerical quantification of the total antibodies within only 7 min from a single drop of capillary blood. We demonstrated a high level of correlation between the POC and the two CLIA laboratory-based immunoassays from Diasorin, thus allowing a potentially wider use of quantitative serology tests in the COVID-19 pandemic.

## INTRODUCTION

During the coronavirus disease 2019 (COVID-19) pandemic, anti-severe acute respiratory syndrome coronavirus 2 (anti-SARS-CoV-2) serological testing has been shown to play an important role not only as a diagnostic support tool but also in understanding antibody responses mounted upon SARS-CoV-2 infection and vaccination (1–3).

The spike (S) glycoprotein of SARS-CoV-2 forms surface-exposed homotrimers that mediate viral entry into host cells. Spiked glycoprotein is therefore the main target of SARS-CoV-2-specific neutralizing antibodies upon infection and the focus of therapeutic and vaccine designs (4–8). The correlates of protection are based on the specific level of SARS-CoV-2-specific neutralizing antibodies, acquired through vaccination or natural infection, that substantially reduces the risk of (re)infection (9, 10).

In clinical trials, antibody production and cellular T cell responses have been measured for these candidate vaccines (11–15). It has been shown that a large proportion of the individuals who mount immunoglobulin G (IgG) antibody responses against the viral S protein generate detectable neutralizing antibody responses (9) and that S protein binding assays correlate significantly with neutralization of wild-type SARS-CoV-2 virus (16–22). Among the different subunits, the S protein in its trimeric form, when used in serology assays, has a high sensitivity (23) and specificity (22).

Quantification of antibody responses and conversion rates of vaccinated populations can provide useful information not only to estimate the variety of vaccine responses and duration of protection but also to enhance vaccine immunogenicity, dosage optimization, amount, and time intervals (6, 24). Therefore, it is inevitable that SARS-CoV-2 S-based assays play an essential role in vaccine efficacy monitoring.

Several quantitative IgG or total antibody tests based on enzyme-linked immunoassay (ELISA) or chemiluminescence-based instruments (CLIA) have been commercialized, and their performances have been evaluated in depth (25–27). However, none of these methods are applicable for antibody quantification in decentralized settings. Standardization of the First WHO International Standard for anti-SARS-CoV-2 immunoglobulin (human; NIBSC code 20/136) has been introduced to allow for comparability between assay results. The International Standard is based on pooled human plasma from convalescent patients, which is lyophilized in ampules, with an assigned unit of 250 international units (IU) per ampule for neutralizing activity. For binding assays, a unit of 1,000 binding antibody units (BAU) per milliliter can be used to assist in the comparison of assays detecting the same class of immunoglobulins with the same specificity (28).

The threshold of protection for anti-SARS-CoV-2 S protein antibodies acquired by vaccination is an object of research in the recent phase of the pandemic. Initial studies show that antibody levels associated with immunity against symptomatic COVID-19 infection measure about 150 to 200 BAU/mL, using the WHO International Standard (10, 29, 30). High antibody titers have been reported as above 250 BAU/mL (31). Recent studies show correlations among antibody titers 1 month postvaccination with the occurrence of breakthrough infections (32).

A third vaccine shot has been shown to boost immune systems and block new emerging coronavirus variants (33).

The aim of this study is to evaluate the performance of a new rapid quantitative point-of-care commercially available device from BluSense Diagnostics, based on the SARS-CoV-2 trimeric spike protein, ViroTrack Sero COVID-19 Total Ab, with two CLIA laboratory-based immunoassays from Diasorin, LIAISON SARS-CoV-2 S1/S2 IgG and LIAISON SARS-CoV-2 Trimeric S IgG assay. LIAISON SARS-CoV-2 Trimeric S IgG was chosen as comparison as it utilizes the same antigen of the ViroTrack test. The performance of the quantitative POC technology was evaluated on capillary and venous blood drawn from 101 hospital-employed volunteers. A panel of precharacterized negative plasma and serum was further evaluated to determine the assay specificity.

## RESULTS

### Participant characteristics.

A total of 101 participants were included. All characteristics can be found in Table 1. A total of 47.5% of participants were between 20 and 39 years old, 44.65% were between 40 and 59 years old, and 8% were over 60 years of age. Of 101 participants, 93 had received two doses of vaccine, 1 had received one dose, and 7 were unvaccinated. Out of the double-vaccinated participants, 52 received their second dose between 2 and up to 5 weeks after the first dose, and 41 participants received their second dose at least 5 weeks (and up to 12 weeks) after the first dose. All the participants who received ChAdOx1 nCoV-19 (Astra Zeneca) as the first dose received BNT162b2 mRNA (Pfizer-BioNTech) as their second dose after at least 10 weeks. At the time of the study, all the participants who received BNT162b2 mRNA had received their second dose between 13 weeks to 29 weeks before the study, and all the participants who received ChAdOx1 nCoV-19 had received their second dose between 3 to 7 weeks before the study.

**TABLE 1:** Characteristics of the study subjects^*a*^

Characteristic	Value for subjects
Age	41 (23–67)
Gender	
Female	89 (88%)
Male	12 (12%)
Body mass index, kg/m^2^	23 (19–40)
Immunosuppressive disorder	3 (3%)
Immunosuppressive medication^*b*^	10 (10%)
Smoking	3 (3%)
Occupation^*c*^	
Nurse	21 (21%)
Junior doctor	22 (22%)
Senior doctor	5 (5%)
Physiotherapist	27 (27%)
Secretary	10 (10%)
Other	17 (17%)
Prior infection with SARS-CoV-2	
Once	26 (26%)
More than once	0 (0%)
Days from infection to test (median)	191 (IQR 20–464)
Vaccinated against SARS-CoV-2	94 (93, 1%)
Prior infection with SARS-CoV-2 and vaccinated	21 (21%)
Never infected with SARS-CoV-2 and not vaccinated	2 (2%)
If vaccinated, first vaccine received^*d*^	
* *BNT162b2 mRNA (Pfizer-BioNTech)	87 (86%)
* *ChAdOx1 nCoV-19 (AstraZeneca)	7 (7%)
Days from injection to test (median)^*e*^	
First injection	169 (IQR 32–196)
Second injection^*f*^	136 (IQR 21–155)

a All values are median and interquartile range.

b Includes systemic and topical medicine (e.g., steroid nasal spray or inhalation).

c One study subject identified with two occupations.

d All vaccinated participants had received Pfizer-BioNTech (Comirnaty) as their second injection regardless of type of the first.

e The day of test was noted as the last study day resulting in an uncertainty of 0 to 3 days.

f One study subject was yet to have the second injection when participating.

One capillary blood sample was not collected for one participant.

### Comparison of assay performances in plasma.

Plasma was analyzed by the POC device and the central lab CLIA-based assays. All vaccinated individuals had positive antibody titers with the ViroTrack system and the two Diasorin IgG assays. Two participants who were not vaccinated but had been previously infected by COVID-19 were negative by Diasorin TrimericS IgG but low positive (20 to 50 BAU/mL) with ViroTrack for all specimen types.

**FIG 1:**
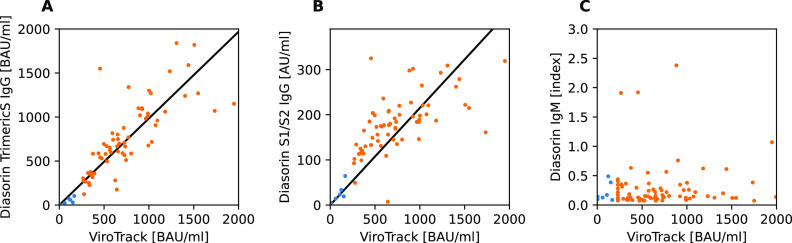
LIAISON SARS-CoV-2 Trimeric IgG (A), S1/S2 IgG (B), and IgM (C) versus ViroTrack Sero COVID-19 Total Ab. For ViroTrack Sero COVID-19 Total Ab, results are shown for diluted plasma (orange points) at >230 BAU/mL; otherwise, undiluted results are shown (blue points). Results inside the dynamic range for Diasorin (<2,080 BAU/mL for Trimeric IgG and <400 AU/mL for S1/S2 IgG) and below 2,000 BAU/mL obtained for ViroTrack are shown.

Figure 1 shows the correlation between LIAISON SARS-CoV-2 S1/S2 IgG, LIAISON SARS-CoV-2 TrimericS IgG assays, LIAISON SARS-CoV-2 IgM, and ViroTrack Sero COVID-19 Total Ab for plasma samples.

A strong correlation between ViroTrack and the Diasorin TrimericS IgG and Diasorin S1/S2 IgG was observed. The Spearman rank’s correlation coefficient was above 0.83 for all methods (Table 2), and all the *P* values were below 10^−26^. The highest correlation with ViroTrack was by the Diasorin Trimeric S assay. The Diasorin M assay did not correlate with the other tested methods.

**TABLE 2:** Spearman rank’s correlation coefficient; 16 samples above the dynamic range for Diasorin (≥2,080 BAU/mL) are excluded from the analysis

Assay	Spearman rank’s correlation coefficient for assay:			
	ViroTrack	Diasorin TrimericS	Diasorin S1/S2 IgG	Diasorin IgM
ViroTrack	1.00	0.89	0.83	0.11
Diasorin TrimericS		1.00	0.93	0.14
Diasorin S1/S2 IgG			1.00	0.12
Diasorin M				1.00

### Comparison among capillary blood, venous blood, and plasma results.

**FIG 2:**
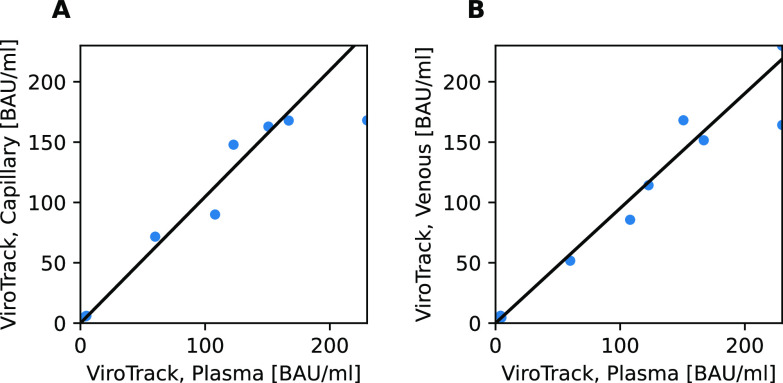
Specimen type agreement between ViroTrack Sero COVID-19 Total Ab results, using capillary blood, venous blood, and plasma samples. (A) Undiluted capillary blood versus undiluted plasma samples. (B) Undiluted venous blood versus undiluted plasma samples.

Having established the correlation between ViroTrack and the reference test methods, we investigated the correlation between different specimen types. Figure 2A and B show the results for different undiluted specimen types for values below 230 BAU/mL.

The correlation between the different specimen types measured by ViroTrack and the plasma samples using Diasorin TrimericS IgG for determination of above 230 BAU/mL is shown in Table 3. The agreements are above 90% between all specimen types and both methods.

**TABLE 3:** Agreement between specimen types and methods of determination of >230 BAU/mL in undiluted samples; values in the parentheses are 95% confidence intervals calculated using the Clopper-Pearson method

Specimen type	Agreement for method of determination:			
	ViroTrack, plasma	ViroTrack, capillary blood	ViroTrack, venous blood	Diasorin TrimericS IgG
ViroTrack, blood plasma	100.0% (96.4%, 100.0%)	99.0% (94.6%, 100.0%)	99.0% (94.6%, 100.0%)	92.0% (84.8%, 96.5%)
ViroTrack, capillary blood		100.0% (96.4%, 100.0%)	98.0% (93.0%, 99.8%)	93.0% (86.1%, 97.1%)
ViroTrack, venous blood			100.0% (96.4%, 100.0%)	91.0% (83.6%, 95.8%)
Diasorin TrimericS IgG				100.0% (96.4%, 100.0%)

### Quantitative results versus vaccination/previous infectious status.

**FIG 3:**
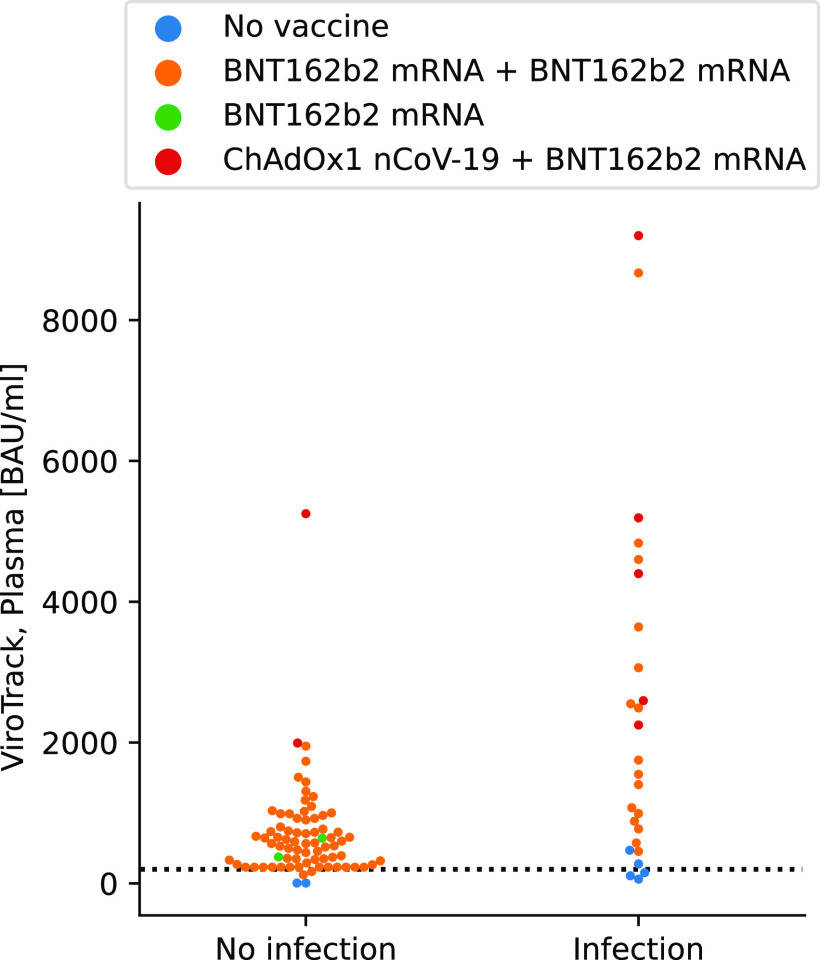
Swam plot of plasma and diluted plasma measured with ViroTrack Sero COVID-19 Total Ab. Data divided into previous PCR confirmed infected (Infection) and previous noninfected (No infection). The dotted line shows 230 BAU/mL.

The quantitative results obtained by the POC device were analyzed according to vaccination and prior infection. Figure 3 shows a swarm plot of the data. For the nonvaccinated, previously infected, the median was 1,850.8 BAU/mL (interquartile range [IQR] 59.9; 3,641.7). For the non-previously infected who were vaccinated (two injections), we observe a lower antibody response (median: 2,686.8 BAU/mL [IQR 122.8; 5,250.9]) compared to that of the previously infected and vaccinated (two injections) participants (median: 4,827.3 BAU/mL [IQR 454.6; 9,200]). In general, we observed a higher antibody response for participants with ChAdOx1 nCoV-19 as the first dose and BNT162b2 mRNA as the second dose (previously infected and vaccinated: *n* = 5, median: 5,724.5 BAU/mL [IQR 2,249.1; 9,200]; only vaccinated: *n* = 2, median: 3,622.1 BAU/mL [IQR 1,993.3; 5,250.9]) compared to that for participants receiving both doses with BNT162b2 mRNA (previously infected and vaccinated: *n* = 15, median: 4,562.9 BAU/mL [IQR 454,6; 8,671.3]; only vaccinated: *n* = 70, median: 1,036 BAU/mL [IQR 122.8; 1,949.2]). However, these participants had all received the second dose with BNT162b2 mRNA less than 50 days before the study, whereas the participants receiving two doses of BNT162b2 mRNA in most cases received the second dose more than 125 days before the time of the study. The time between the injections also varied for the two groups.

Several of the noninfected who received both doses of BNT162b2 mRNA showed values below 230 BAU/mL. For the previously infected, only participants without any vaccination had values below 230 BAU/mL. The capability of the assays to correctly quantify a high positive sample (e.g., >230 BAU/mL) can be important, as revealed by recent reports correlating between the antibody levels and the immunity against symptomatic COVID-19 infection (29, 30).

The study included two nonvaccinated and noninfected participants (see Table 1). The two subjects tested negative by all methods and sample types. To determine the specificity of the assay, a panel of 699 negative samples were measured. It was composed of samples from healthy donors, samples collected prior to 2019, and samples positive for other viruses, bacteria, or potential cross-reactive substances. Data are reported in Table 4 and show a specificity of 99.7%.

**TABLE 4:** Negative sample panel, plasma and serum, tested during the validation process of ViroTrack Sero Covid Total Ab; a total specificity of 99.7% is obtained over a total of 699 samples^*a*^

Donor status/condition	No. of samples that tested:			*n*	Specificity
	NEG	EQV	POS		
Healthy donor	246	4	1	251	99.6%
Healthy pregnancy	5			5	100.0%
Influenza	11			11	100.0%
Other human coronavirus	20			20	100.0%
Respiratory infection/human rhinovirus/enterovirus/RS virus/parainfluenza virus 1–6/human metapneumovirus (hMPV)	17			17	100.0%
Epstein-Barr virus/herpes simplex virus (HSV)/HIV	25			25	100.0%
Borrelia/leishmaniasis/syphilis/toxoplasmosis	24			24	100.0%
Cytomegalovirus/rubella antibody/varicella-zoster virus	19			19	100.0%
Rheumatoid factor/HAMA	20			20	100.0%
Chlamydia pneumoniae/Legionella pneumophila/Mycoplasma pneumoniae	29			29	100.0%
Hepatitis A+B+C virus (HCV)	24		1	25	96.0%
Pneumocystis jirovecii (PJP)/lupus human serum/Mycobacterium tuberculosis	14			14	100.0%
Streptococcus pneumoniae/Streptococcus pyogenes/Haemophilus influenzae b/Bordetella pertussis	13			13	100.0%
Antinuclear and anti-mitochondrial antibodies	10			10	100.0%
Chagas/chikungunya/dengue/malaria/West Nile/Zika	72			72	100.0%
Unknown	143	1		144	100.0%
					
Grand total	692	5	2	699	99.7%

a NEG, negative; EQV, equivalent; POS, positive.

## DISCUSSION

To our knowledge, this is the first report comparing the numerical results of a rapid quantitative COVID-19 serology test with a reference CLIA method. There exist a few quantitative rapid serology tests with a reader based on fluorescence lateral flow tests already; however, so far, only qualitative rapid tests have been evaluated in literature (34). Our study showed a statistically high level of correlation between the results from ViroTrack Sero COVID-19 Total Ab and those from the two CLIA laboratory-based immunoassays from Diasorin, LIAISON SARS-CoV-2 S1/S2 IgG and LIAISON SARS-CoV-2 Trimeric S IgG assay. The highest correlation, 0.94, was found for LIAISON SARS-CoV-2 Trimeric S IgG assay, which utilizes the same spike trimer antigen as ViroTrack Sero COVID-19 Total Ab.

Previous reports of other commercial spike protein-based IgG assays (ELISA or CLIA based) showed a lower level of correlation of around 0.7 to 0.8 (26) with or without standardization to BAU/mL units (25). The high correlation was obtained even though the POC method measures total antibodies (IgG, IgM, and IgA) while the reference methods measured only IgG and/or receptor binding domain. The low influence of IgA and IgM antibodies may be explained by a low IgM concentration, a general correlation between IgA and IgG titers, or the predominance of IgG antibody class in vaccinated individuals.

The agreement among specimen types was satisfactory. As described, the ViroTrack Sero COVID-19 Total Ab assay is embedded into a centrifugal microfluidics platform where blood is separated into plasma in the initial processing steps. This unique capability allows for the precise quantification without influence of factors such as hematocrit, enabling precise correlation with laboratory-based methods. To our knowledge, systematic studies comparing COVID-19 antibodies in different matrixes do not exist; however, preliminary studies show differences in rapid test results when capillary or venous blood is used (35).

We observed a higher antibody response for participants with ChAdOx1 nCoV-19 as the first dose and BNT162b2 mRNA as the second dose less than 50 days prior to the study compared to that of the participants who, in most cases more than 125 days prior to the study, received their second dose of their two doses from BNT162b2 mRNA. Furthermore, its known that heterologous boosting results in higher titers than homologous boosting (36, 37).

A limitation of this study is represented by the fact that only two individuals were neither previously infected nor vaccinated; however, the large negative retrospective sample panel measured (699 samples) confirmed the high specificity of the product. In addition, in a previous study we showed that a first version of the POC test targeting the antibodies against SARS-COV-2 nucleocapsid protein had a higher specificity than ELISA-based methods (38). Second, the study is limited, as an extra dilution step was necessary to extend the current dynamic range of the POC device which is currently not included in the product “instructions for use,” and the dilution process performed in blood may have produced a different result. However, the data demonstrated that the device produced an accurate quantification of diluted plasma.

A general agreement between capillary blood, venous blood, and plasma from the same samples and techniques has been found, thus supporting the use of capillary blood on the POC device for precise decentralized antibodies monitoring postvaccination and responses after natural infection in countries where the use of vaccines is low or yet to come.

Among the vaccinated-only individuals, 17 (18%) had antibodies below 200 BAU/mL, but their median time from last vaccination to antibody did not differ from that of patients who had above 230 BAU/mL (136 days). This could be caused by several things, including unrecognized or undiagnosed infection with COVID-19, as none of our assays included antibodies against antigens/epitopes other than the spike protein and information on previous infection with SARS-CoV-2 relied solely on participant memory, which could introduce a bias. On the other hand, the main purpose of the study was to evaluate the performance of a new technology and our result should not be affected by this.

In conclusion, ViroTrack Sero COVID-19 Total Ab provides an accurate numerical quantification of the total antibodies against the spike protein trimer within 7 min from a single drop of capillary blood. Compared to rapid lateral flow tests detecting antibodies against different forms of the spike protein, the evaluated POC device provides a numerical result in a shorter time.

This capability can enable precise monitoring of antibodies amounts in facilities in various places, allowing a potentially wider use of quantitative serology tests in the COVID-19 pandemic.

## MATERIALS AND METHODS

### Subjects and samples.

All participants were staff, and included individuals were from most types of professions (e.g., doctors, nurses, physiotherapists, cleaning staff, etc.). Each study subject was asked to fill in a questionnaire using research electronic data capture (Table 1). The study was approved by the Regional Ethics Committee of the Capital Region of Denmark (record no. H-20046624). The study was further approved by the Regional Data Protection Center (record no. P-2020-358).

### Sample collection.

Capillary and venous blood samples were collected from each of the study subjects. Venous blood, capillary blood, and plasma samples (diluted or undiluted) were analyzed with the ViroTrack Sero COVID-19 Total Ab. The plasma samples were further analyzed on a Diasorin LIAISON XL analyzer at the Department of Clinical Microbiology. Approximately 5 mL of venous blood was collected per subject in a blood collection tube (BD Vacutainer, UK) treated with potassium ethylenediaminetetraacetic acid (EDTA). Blood was processed following manufacturer’s instruction and used for plasma separation. Briefly, blood was centrifuged for 15 min at 1,500 × *g* and 20°C to obtain plasma.

For analyzing capillary blood, 20 μL was collected with a micropipette or a capillary pipette.

Capillary blood was loaded immediately onto the ViroTrack Sero COVID-19 Total Ab cartridge after collection; product specifications allow for 3 min before starting the test. Venous blood was stored at room temperature after collection and to a maximum of 5 h before testing on the ViroTrack Sero COVID-19 Total Ab cartridge. Plasma was separated and stored at room temperature and to a maximum of 5 h before testing onto the ViroTrack Sero COVID-19 Total Ab cartridge. All plasma samples were stored at −80°C prior to Diasorin LIAISON XL analyzer testing, which took place 7 to 11 days after the study. To assess the specificity of the test, a panel of 699 negative plasma and serum samples were tested at BluSense Diagnostics as part of the product validation and CE marking certification process. The samples were previously characterized by the sample providers and/or collected before 2019.

### ViroTrack Sero COVID-19 Total Ab.

The ViroTrack Sero COVID-19 Total Ab is a POC rapid test providing quantitative results within 7 min in the range of 10 to 230 BAU/mL from 20 μL blood, plasma, or serum. The test format is composed by a cartridge (ViroTrack Sero COVID-19 Total Ab) and a reader (BluBox).

The platform utilizes a centrifugal microfluidic platform together with an optomagnetic readout based on the agglutination of magnetic nanoparticles (IMA). In brief, 20 μL of sample is loaded on to the microfluidic cartridge, which is then inserted inside the reader (BluBox). In the case of whole blood, the red blood cells are separated from the plasma by centrifugal force. The separated plasma is subsequently resuspended in the prestored reagents on the cartridge (e.g., magnetic particles). The magnetic particles are functionalized with SARS-CoV-2 trimeric spike protein and agglutinates in a sample containing anti-spike antibodies. Incubating the particles in a homogeneous magnetic field speeds up the reaction kinetics of the agglutination (39, 40). For optomagnetic detection, a uniaxial alternating magnetic field is applied which periodically aligns the agglutinated particle chains, which results in a modulation of the transmitted light proportional with the target concentration (41). IMA does not require labeled secondary antibodies (38).

The ViroTrack Sero COVID-19 Total Ab was calibrated to the First WHO International Standard for anti-SARS-CoV-2 immunoglobulin (code: 20/136), and the results were converted to binding antibody units per milliliter (BAU/mL) by the software up to 230 BAU/mL. Plasma samples with >230 BAU/mL were diluted 10 times in phosphate-buffered saline (PBS) and remeasured. Dilution (20 times and 40 times) was continued until a result below 230 BAU/mL was obtained. The final binding antibody units per milliliter was found by multiplying the dilution factor with the obtained result. During the study, four different BlueBox readers were used in parallel. Samples were loaded in different readers in a randomized order.

The same data are shown in the product instruction for use and used for CE certification.

### Diasorin.

The plasma samples were analyzed by chemiluminescence immunoassay (CLIA) for SARS-CoV-2 antibodies (IgM and IgG) targeting the subunits of the spike proteins S1 and S2 and the trimeric spike complex, including the receptor binding domain (S1-RBD). Samples were analyzed on the DiaSorin LIAISON XL analyzer using LIAISON SARS-CoV-2 IgM, LIAISON SARS-CoV-2 S1/S2 IgG, and LIAISON SARS-CoV-2 TrimericS IgG assays in accordance with the manufacturer’s instructions on the specific assay. A negative anti-SARS-CoV-2 result was defined as IgM index of <1.1 and IgG of <12 antibody units (AU)/mL, and a positive result was defined as an index value of ≥1.1 AU/mL for IgM and ≥15 AU/mL for IgG, respectively. Results for IgG of <15 AU/mL and ≥2 AU/mL were reported as inconclusive.

A TrimericS IgG result was defined as negative with a value of <13 AU/mL, and a positive result was defined as values of ≥13 AU/mL (equivalent to ≥33.8 BAU/mL). The LIAISON SARS-CoV-2 TrimericS IgG assay measures between 4.81 and 2,080 BAU/mL. A recent study demonstrated that the DiaSorin SARS-CoV-2 S1/S2 IgG antibodies had a sensitivity of 96.2% and a specificity of 98.9% (42), whereas the DiaSorin TrimericS IgG has been shown to have a higher sensitivity of 99.4% and a higher specificity of 99.8% (22).

### Statistical analysis.

Spearman’s rank correlation was measured to evaluate the agreement between the different assays and the different specimen types. Analysis and graphs were performed using PYTHON/R.
